# Postural sway dynamics in adults across the autism spectrum: a multifactor approach

**DOI:** 10.1186/s13229-025-00676-y

**Published:** 2025-08-28

**Authors:** Bess F. Bloomer, Amanda R. Bolbecker, Emily L. Gildea, Daniel P. Kennedy, Krista M. Wisner, Brian F. O’Donnell, William P. Hetrick

**Affiliations:** 1 Psychological and Brain Sciences, Indiana University, Bloomington, IN USA; 2 Joint Doctoral Program in Clinical Psychology, San Diego State University/UC San Diego, 6363 Alvarado Ct. Suite 200, San Diego, CA 92120 USA; 3 Program in Neuroscience, Indiana University, Bloomington, IN USA; 4 Department of Psychiatry, Indiana University School of Medicine, Indianapolis, IN USA; 5 Psychological and Brain Sciences, University of Iowa, Iowa City, IA USA

**Keywords:** Autism, Postural sway, Postural control, Multiple factor analysis, Rambling, Trembling, Sway patterns, Adults

## Abstract

**Background:**

Motor challenges are highly prevalent within autism, and increased postural sway has been consistently demonstrated in autistic youth. However, the extent to which sway anomalies extend into adulthood remains understudied. This study aimed to investigate whether increased postural sway is altered in autistic adults compared to neurotypical controls using established sway metrics including sway area and path, as well as rambling-trembling decomposition—an approach that differentiates the postural sway signal into central and peripheral nervous system components.

**Methods:**

49 adults with autism spectrum conditions (ASC) and 94 neurotypical controls (NC) participated in a postural sway task on a force platform with manipulations of visual input and stance width. Traditional geometric methods (sway area and path), the spatial characteristics of the body’s adjustment to maintain balance, were measured. As resulting sway measures often covary, multiple factor analysis (MFA) was applied to reduce the measures into distinct, non-redundant dimensions that simplified the data. Group comparisons were completed across these different levels of analysis.

**Results:**

We observed increased sway path and medio-lateral trembling in ASC compared to NC (*p* < 0.05). Significant group by vision interactions revealed that ASC sway increases were more apparent in eyes-open conditions for sway area and rambling and trembling in the anterior-posterior plane (*p* < 0.01), possibly suggesting differential sensory reweighting of visual input by ASC, or difficulties with multisensory integration. MFA revealed two key dimensions. A fast frequency dimension, linked to peripheral processes, explained most of the overall variance (62.9%) and captured more variance in the ASC group than in NC. A slower frequency dimension, linked to central processes, explained 8.0% of the variance.

**Limitations:**

Order of sway conditions was consistent among all participants, so it is possible that participant fatigue influenced later sway conditions.

**Conclusions:**

Building upon previous research finding increased postural sway in autism, we found that combining multiple approaches collectively suggest the critical role of peripheral contributions and visual input in postural sway in autism. Fast-frequency processes that are peripherally-driven may be of particular importance in sway in autistic adults, and should be prioritized in future research to better understand balance performance in autism.

**Supplementary Information:**

The online version contains supplementary material available at 10.1186/s13229-025-00676-y.

## Introduction

Autism is a neurodevelopmental condition characterized by diverse challenges, including differences in social interaction, communication, and restricted and repetitive behaviors, with symptoms varying widely in presentation across individuals [1]. Although autism presents with high clinical heterogeneity, challenges in sensorimotor domains are common, with motor problems affecting approximately 85% of autistic individuals [2, 3]. Motor differences in autistic children have been well-documented. Previous literature has consistently found greater postural instability [4–7], and other gait, balance, and coordination difficulties [8, 9] in children with autism compared to neurotypical youth.

The maintenance of upright and stable posture is a dynamic and vital process which enables humans to carry out functions and activities essential to daily living [10]. Postural control requires the interplay of multiple, separable systems that utilize different neurological components operating on different timescales. Within the central nervous system (CNS), vestibular and visual system contributions to postural control occur at low temporal frequencies (~ 0-0.5hz) [11, 12]. The peripheral nervous system (PNS) contributes reflexive corrections mediated by fast spinal motoneuron loops that operate at high temporal frequencies (> 1-2hz) and are driven by proprioceptive input [12, 13].

Measurement of postural sway involves quiet standing on a force platform which collects corrective movements made to maintain the center of gravity, resulting in a time series representing adjustments of what is termed the center-of-pressure (CoP). Sway path (the length travelled by the CoP), and sway area, (the area covered by the CoP) are traditional measures of steadiness and stability during standing postures, and have been shown to be robust in differentiating between clinical groups [14–16]. An important goal is to dissociate and quantify the relative contributions of different nervous system mechanisms that control postural sway. One method of delineating postural sway that has garnered substantial empirical support is the rambling-trembling analysis of postural sway proposed by Zatsiorsky and Duarte [17]. The rambling-trembling method decomposes the CoP signal into (i) a rambling component representing a centrally defined, migrating reference point generated by the CNS, and (ii) a trembling component, defined as the fluctuations around this migrating reference point that are thought to represent PNS contributions to sway. Empirical evidence supports the existence and separability of these two components. For instance, rambling and trembling change independently from one another in response to task demands [18, 19].

Covariance among postural sway metrics is high, which can make interpretation difficult. Moreover, many studies use multiple sway metrics, which raises the issue of multiple comparisons and the concern that applying corrections could obscure true differences. One approach to address this conundrum is to reduce the set of variables into a smaller number of independent dimensions using Multiple Factor Analysis, or MFA [20]. MFA can be used to study groups of variables collected for the same set of individuals and can incorporate both categorical and continuous variables in defining dimensions, (or components as they are commonly called in Principal Components Analysis (PCA), which is a related approach). For the purposes of the present study, MFA was chosen because it is an approach akin to performing a repeated measures PCA that can assess the effects of altering visual input and stance. Critically, MFA is a multivariate statistical approach that identifies highly correlated variables likely to be influenced by a common factor and therefore can identify postural sway metrics that measure similar features of sway. PCA/MFA approaches have not been applied to postural sway in autism samples, resulting in a lack of knowledge regarding how postural sway metrics are related to each other and whether components that account for the most variability in the sway signal differ across groups.

In addition to past literature supporting greater postural instability in autistic children, some reports suggest increased postural sway is correlated with behavioral symptoms of autism including restricted and repetitive behaviors [21–25]. Increased postural sway has also been shown to negatively impact autistic youth in a variety of non-motor domains, through interference with the development of fine motor skills as well as efficient coordination of social interactions [26–28]. Considering the consequences of increased postural sway in a variety of non-motor activities of daily life [26–28], more thorough identification of underlying processes contributing to postural instability could allow for the development of more targeted treatments which could improve overall quality of life for older autistic adults. As motor symptoms are core features of autism throughout the lifespan [29–31], postural sway is a critical yet fundamental motor task that can be used to probe the integrity of motor circuits [32, 33]. Moreover, rambling and trembling methods within postural sway delineate specific nervous system abnormalities [17, 34], but have been scarcely studied in autistic adults. Despite evidence supporting differences in postural sway and motor stability in youth with autism, and the known effect of development and aging in postural control [35, 36], postural sway within autistic adults remains understudied. A handful of recent studies examined postural sway development and general motor signatures along the autism spectrum and showed evidence that postural sway differences remain in autistic adults [7, 23, 37], as well as neurotypical adults endorsing high levels of autistic traits [38], and that autism symptom severity was moderately correlated with the severity of motor outcomes [31]. Taken together, a better understanding of the neurological mechanisms underlying postural stability in autistic adults is necessary for not only knowing how neural systems differ within this population, but also understanding how sensorimotor symptomatology presents in adulthood.

In the current study, we compared postural sway metrics in adults with autism spectrum conditions (ASC) to neurotypical comparison participants (NC), in the context of visual and proprioceptive manipulations using both traditional geometric measures (sway area and path length) and rambling-trembling analyses. MFA was then used to simplify these metrics, identify which approaches measure similar or unique processes, and explore how these patterns differ in autistic adults. First, we hypothesized there would be increased postural sway in geometric measures (sway area and path) within the ASC group versus the NC group, with larger differences in conditions where visual or proprioceptive inputs were challenged (e.g. Eyes Closed and/or Base Closed conditions) in line with previous literature [7, 39, 40]. Due to the relative lack of literature incorporating rambling-trembling decomposition analysis into studies of postural sway and autistic adults, we hypothesized overall differences between groups on rambling/trembling without specifying directionality.

## Methods

### Participants

Participants with ASC and neurotypical comparison participants (NC) between 18 and 50 years of age were recruited from Indiana University and the surrounding communities of Bloomington and Indianapolis via physical flyers and social media advertisements to take part in a larger study assessing biobehavioral markers within autism. Written consent was obtained from all participants. All study procedures were approved by the Indiana University Institutional Review Board and conducted in accordance with the Declaration of Helsinki.

The NC group was composed of non-clinical volunteers. The ASC group was recruited using advertisements specifically inviting individuals in the autism community to participate who self-identified with the diagnosis. During phone screening conducted by trained staff, if a past professional diagnosis of autism (i.e.: autism spectrum disorder, Asperger’s syndrome, PDD-NOS) seemed likely, the individual was invited to enroll in the study. Further assessment of enrolled participants included clinical interviews based on DSM-V criteria for autism spectrum disorder and questionnaires. All participants were assessed by the Structured Clinical Interview for the DSM-5 (SCID 5-RV) [41] and a related assessment of personality disorders [42], as well as the Wechsler Abbreviated Scale of Intelligence 2nd Edition (WASI-II) [43].

Self-report questionnaires to characterize the ASC sample included the Autism Spectrum Quotient (AQ-Full) [44], Broad Autism Phenotype Questionnaire (BAP-Q) [45], and the Social Responsiveness Scale (SRS) [46]. Clinical cut-off scores are represented by a clinical threshold of ≥ 32 total and a screening cut-off of ≥ 26 total on the AQ [44], clinical cut-offs of ≥ between 3.0 and 3.75 average item scores for the whole measure and each subscale for the BAP-Q [45], and a cut-off total score of ≥ 75 on the SRS [46]. Participants in the ASC sample had scores on these measures that met, or in most cases, exceeded clinical cut-off thresholds but were generally on the lower end of these ranges (See Supplementary Materials). Further, they had high average cognitive functioning, as indicated by group mean WASI IQ scores around 116, compared to 113 in NC (see Table 1). Sixteen parents of ASC participants also agreed to fill out a lifetime-version of the Social Communication Questionnaire (SCQ) [47] about their child’s lifetime experience of symptoms, thinking patterns, or behaviors relating to autism. For a subset of participants, the Autism Diagnostic Observation Schedule, Version 2 (ADOS-2) [48] was also completed by 11 out of 49 ASC participants). When possible, verification of ASC diagnosis was made using psychiatric records (24 out of 49 ASC participants). Participants in the NC group were required to have no history of psychiatric or neurodevelopmental disorders.

**Table 1:** Subject demographics and variables of interest for full sample

	ASC - Mean (SD) or %	NC - Mean (SD) or %	Statistic	*p*
Age	23.38 (5.66)	22.61 (3.87)	*t*=-1.70	0.08
Sex (% Male)	40.00%	47.87%	*x*^*2*^ = 3.00	0.09
Race (% White) ^§^	89.80%	60.82%	*x*^*2*^ = 12.00	< 0.001**
WASI-II IQ	116.40 (13.32)	113.20 (10.49)	*t* = 2.90	0.004**
Weight (kg)	79.55 (26.67)	69.42 (17.36)	*t =* 4.80	< 0.001**

Total sample was *N* = 143 participants, 49 ASC and 94 NC. ASC, autistic adults; NC, neurotypical control; SD, standard deviation, WASI-II, Wechsler abbreviated scale of intelligence, second edition; IQ, intelligence quotient. Table 1 depicts % White only; further racial breakdown of the sample is as follows: ASC who are non-White included 2% American Indian or Alaskan Native, 0% African American, 4% Asian, 9% More than one race, 0% Unreported/other; NC who are non-White included 8% African American, 19% Asian, 9% more than one race, 3% Unreported/other. *= *p* < 0.05; **= *p* < 0.01

Exclusion criteria for all participants included (a) history of loss of consciousness greater than 10 min duration; (b) presence of any neurological disorders; and (c) WASI-II IQ < 80; (d) current consumption of > 14 alcoholic drinks per week. Additional exclusion criteria for NC participants included current or past psychiatric disorders, and past or current use of psychiatric medications (i.e.: SSRIs, antipsychotics). Additional exclusion criteria for ASC participants included (a) severe psychopathology (i.e. major depression disorder, bipolar disorder, or psychotic disorder); and (b) current use of substances such as cannabis, amphetamines, cocaine, barbiturates, and opiates (as assessed by presence on a urine drug screen). Importantly, because of the high number of adults with autism having co-occurring psychiatric disorders [49–51], participants who met criteria for autism were included even when other psychiatric disorders (e.g. anxiety, attention deficit hyperactivity disorder) were present at lower levels of severity.

After the screening process, 144 (ASC = 49, NC = 94) participants were eligible for further analysis for the current study. See Table 1 for demographics characterization. Of the 49 ASC participants, 28 participants were on psychotropic medication at the time of testing. See Supplementary Materials for clinical characterization of ASC participants, including frequency of co-occurring psychiatric disorders and group mean values from self-report assessments of autism phenotypes.

### Postural sway task procedure

Each participant was required to stand as still as possible while barefoot on an AMTI AccuSway (Watertown, MA) force platform, sampling at 200 Hz. Visual information was manipulated by asking participants to stand with eyes either open or closed. Stance width was manipulated to alter proprioceptive input, as changes in joint position and body orientation directly influence afferent sensory information critical for proprioception [52–55]. Participants stood either with feet together such that they are approximately one inch apart (Closed Base of Support, or BOS) or shoulder-with apart (Open BOS). These sensory manipulations produced four separate conditions for each participant: Eyes Open, Closed BOS (EOCB); Eyes Closed, Closed BOS (ECCB); Eyes Closed, Open BOS (ECOB); and Eyes Open, Open BOS (EOOB). Importantly, the four sway conditions (EOOB, EOCB, ECOB, ECCB) were not randomized due to design limitations, so they were always measured in all participants in the same order. Two-minute recordings of center of pressure (CoP) were made for each condition for each participant.

### Data processing

Matlab R2023a (9.14.0) was used for all data processing [56]. Data were down sampled to 50 Hz using the MatLab function “downsample”, then lowpass filtered (20 Hz cutoff) using a 4th order Butterworth filter.

### Computation of sway area and sway path

Area was computed by fitting a 95% confidence ellipse around axes represented by eigenvalues that were obtained from principal components analysis of the covariance matrix constructed from mediolateral (ML) and anterior-posterior (AP) CoP axes (6000 data points each). MatLab code provided in supplementary materials of Zatsiorsky and Duarte [57] was used for this procedure.

Sway path was calculated as the trajectory of the CoP travelled using the following equation:

$$\begin{array}{l}\:Path\:Length\\=\sum\:_{n-1}^{N}\sqrt{{[x\left(n\right)-x\left(n-1\right)]}^{2}+{\left[y\left(n-1\right)\right]}^{2}\:}\end{array}$$

### Rambling trembling decomposition

Both rambling and trembling were calculated for the ML and AP directions. The CoP trajectory was decomposed into rambling and trembling components following methods described in Zatsiorsky and Duarte [17, 34]. Rambling was computed by determining the instant equilibrium points, in which the horizontal force is zero (indicating perfectly vertical alignment), then using a cubic spline function to interpolate the rambling trajectory. CoP deviations from the instant equilibrium points were used to construct the trembling trajectory. The root mean square was computed for both rambling and trembling trajectories in the ML and AP directions.

### Data analysis

A total of six postural sway measures were computed for each of the 4 sway conditions (Sway area, Sway path, ML rambling, ML trembling, AP rambling, AP trembling). Variables that violated normality (Kolgorov-Smirnof test) were log transformed. Planned comparisons of group differences in these above metrics of postural sway were conducted using mixed ANOVAs for each of the six conditions with Eye and Base as within-subjects factors using the “afex” package in R [58]. Follow-up tests using the emmeans package in R were conducted when significant interactions were present [59]. Post-hoc tests using the Bonferroni correction were conducted when interactions were significant (*p* < 0.05). Since a high percentage of the ASC group was taking psychotropic medication at the time of testing (*n* = 28), and because use of psychotropic medications has been shown to potentially influence postural sway [60], follow-up analyses were conducted between ASC participants on and off psychotropic medications to clarify if medication use influences findings.

### Multiple factor analysis

Multiple Factor Analysis (MFA) is a principal components analysis-based approach used for groups of variables, i.e. sway conditions, composed of multiple measures for each. The FactoMineR [61] package in R was used to conduct multiple factor analysis. MFA is well-suited to repeated measures designs because it can analyse observations for groups of conditions (in this case the four different sway conditions), each with multiple variables (in this case, different sway dependent variables such as area, path, rambling and trembling) and identify their common structures. In MFA, a principal components analysis (PCA) is first run on each condition and the results are then normalized by dividing each element by its first eigenvalue. A global PCA is conducted on the unique matrix formed by the normalized results from each condition. Finally, similarities and differences between conditions are analysed by projecting the individual results from each condition onto results from the global analysis. MFA therefore produces a coherent analysis of the relationships between conditions and the measurements performed for each condition. Factor loadings were computed then rotated using Varimax rotation. In the current analysis, MFA tests were run separately for each group to characterize each group’s specific loading patterns. A final MFA using data from both groups was run to determine between-group differences on key dimensions.

### Confirmation of suitability of data for PCA/MFA

The Kaiser–Meyer–Olkin (KMO) test for sample adequacy was performed including all six sway measurements for each of the 4 conditions separately and confirmed that sample size was sufficient (average KMO > 0.60) [62, 63]. Bartlett’s test for sphericity was also significant for each condition, confirming that correlations between variables were of sufficient size for PCA/MFA.

### Outlier analysis and dimensions considered for interpretation

The R package FactoInvestigate [64] was used to determine outliers for each condition separately. The appropriate number of dimensions to interpret was determined using Horn’s parallel test using the R package paran [65]. For all six sway metrics, “contrib” was computed, which is a measure of the proportion of the variance captured on the MFA dimensions suitable for interpretation.

Rotated values of 0.40 and above were considered substantial [66]. Moreover, to retain focus on significant findings, variables which display a degree of cross-loading (>|0.32|) [67] onto multiple dimensions were only discussed in the context of a variable that has at least one substantial loading (>|0.4).

## Results

### Demographic statistics (Table 1)

The ASC and NC groups did not significantly differ on age or sex assigned at birth (see Table 1). However, the groups did differ on WASI-II IQ scores (2-scale; Weschler, 2011), with the ASC group scoring about three points higher than NC, on average (*t*(322) = 2.90, *p* < 0.01, 95% CI [1.053, 5.376]; see Table 1). When WASI IQ was included as a preliminary covariate in ANOVAs, there were no significant main effects or interactions with group for predicting sway metrics, so it was not included in the final models. Additionally, race was significantly different between groups, with the ASC group being composed of a higher proportion of white participants to non-white participants than NC (*p* < 0.001; see Table 1). Finally, weight was significantly different between groups, with the ASC group having higher body weight on average (*t*(282) = 4.80, *p* < 0.001, 95% CI [5.989, 14.255]; see Table 1). Because weight and race had a significant main effect on sway variables when included as covariates, these demographic variables were kept as covariates in final analyses. Though race was not important to the study’s hypotheses and has not been shown to influence postural sway, race was included as a covariate to reduce potential confounding in group comparisons and to protect against Type I error.

### ANOVA results (Table 2)

After accounting for weight and race, the groups differed significantly on sway path, with the ASC group showing increases on sway path compared to NC (*F*(139) = 4.79, *p* = 0.030). However, the groups did not significantly differ on sway area (*p* > 0.05). There were no significant group by vision or group by Base of Support (BOS) interactions on sway path. For sway area, there was a significant group by vision interaction (*F*(139) = 7.83, *p* = 0.006). Follow-up contrasts showed that both groups exhibited increased sway in eyes-closed conditions compared to eyes-open, but the magnitude of increase was greater in the NC group (*M* difference = 0.36, *p* < 0.0001, 95% CI [0.259, 0.458]) than in the ASC group (*M* difference = 0.17, *p* = 0.009, 95% CI [0.032, 0.310]).

**Table 2:** Mixed ANOVA results for sway metrics between ASC and NC groups

	Group	Eye	Group x Eye	Base	Group x Base	Eye x Base	Group x Eye x Base
Sway Path	4.79*	30.57***	2.46	11.39***	0.91	3.82^†^	0.00
Sway Area	2.71	15.33***	7.83**	23.78***	0.12	4.72*	0.10
AP Rambling	3.06^†^	0.57	8.60**	2.35	1.71	8.06**	1.43
ML Rambling	1.45	10.81**	0.64	20.88***	0.02	2.11	1.07
AP Trembling	0.53	30.99***	3.58^†^	2.73^†^	0.41	0.04	0.24
ML Trembling	4.07*	2.67^†^	1.99	40.52***	3.41^†^	0.04	0.12

The above tables summarize the mixed analysis of variance (ANOVA) findings between the ASC and NC groups on all six sway metrics - sway path, sway area, AP rambling, ML rambling, AP trembling, ML trembling. Significant *p*-values are coded as *p* < 0.10 (trend)^†^; *p* < 0.05*; *p* < 0.01**; and *p* < 0.001***. There was a significant or trending main effect of Group in several sway metrics (sway path, sway area, AP rambling, ML trembling). Additionally, there were significant Group x Eye interactions for sway area, AP rambling, and AP trembling (trend level)

**Fig. 1:**
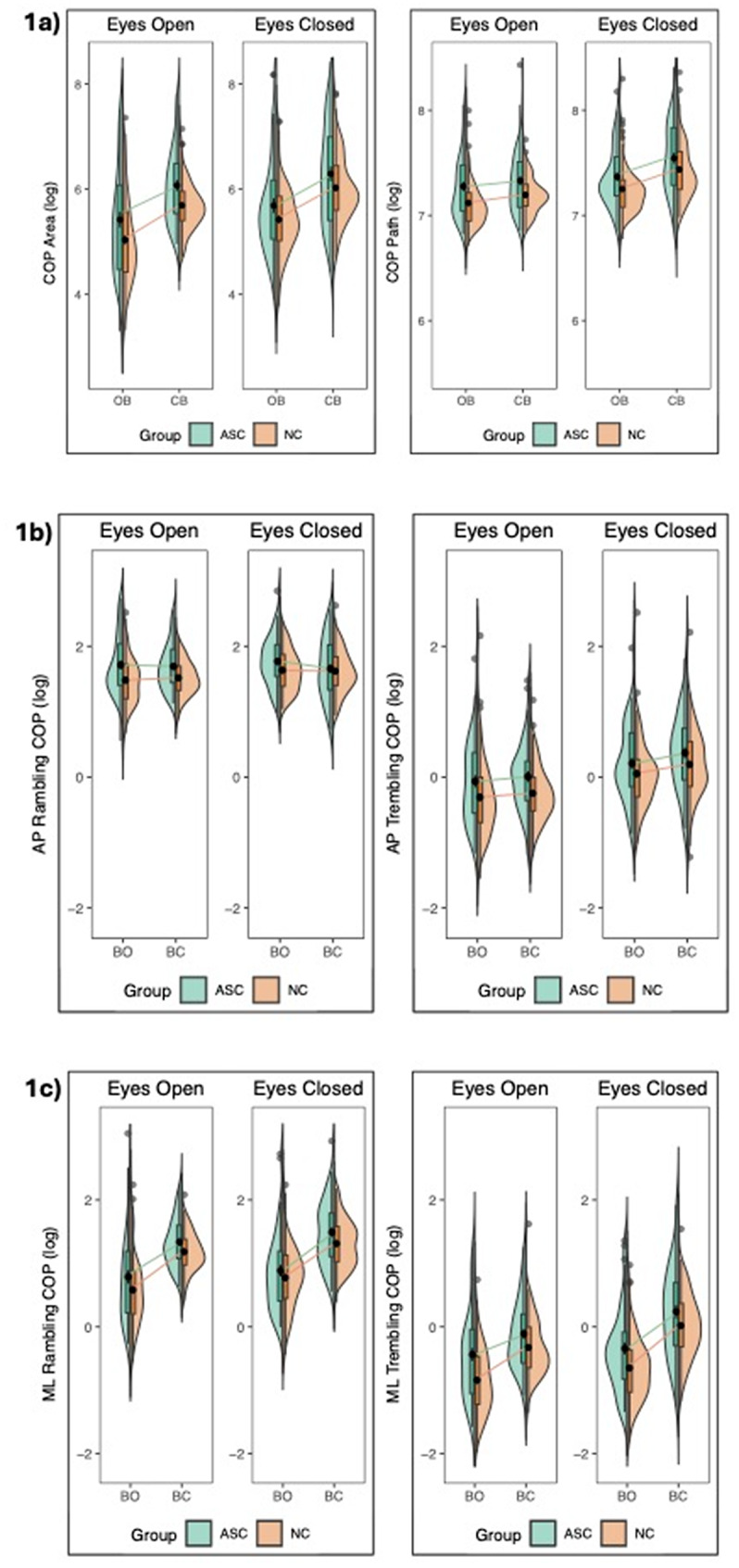
Center-of-pressure (CoP) plots of sway variables for ASC and NC. COP plots for sway variables area and path (Fig. **1a**); ML rambling and ML trembling (Fig. **1b**); AP rambling, AP trembling (Fig. **1c**) for ASC group (green) and NC group (orange). Path length was increased in the ASC group compared to controls (*p* < 0.05; Fig. **1a**). ML trembling was significantly increased in ASC compared to NC (*p* < 0.05; Fig. **1b**). AP rambling and AP trembling were not significantly increased in ASC compared to NC (Fig. **1c**.)

Sway rambling was not significantly increased in ASC compared to NC in either AP or ML directions (*p* > 0.05), directions. AP rambling demonstrated a significant group by vision interaction, such that, though both groups decreased sway when eyes were open, the ASC group had significantly greater AP sway rambling in the eyes-open conditions compared to controls, suggesting lower ability to modulate sway based on increased visual input (*t*(139) = 2.86, *p* < 0.05, 95% CI [0.014, 0.301]; see Table 2). There were no significant group interactions for ML rambling. In the ASC group compared to NC, we also observed significantly increased trembling in the ML direction (*F*(139) = 4.07, *p* = 0.045). Follow-up contrasts showed that the ASC group showed greater ML trembling (adjusted mean = − 0.252) than the NC group (adjusted mean = − 0.415), with a significant group difference of 0.163 (*p* = 0.046, 95% CI [0.003, 0.323]). There were no significant group differences or interactions for AP trembling (*p* > 0.05). See Fig. 1 for CoP plots of a visual depiction of group differences based on these sway variables.

To further clarify findings, we ran ANOVAs on each of the six sway metrics described above when participants were instead categorized across three levels (ASC-medicated, ASC-unmedicated, and NC) to assess potential impacts of medication on postural sway differences. Follow-up tests revealed that there were no significant group differences between ASC-medicated and ASC-unmedicated on any sway metrics. The only significant finding between ASC-medicated and ASC-unmedicated in these ANOVAs was a significant group by BOS interaction on ML rambling, such that in open-base conditions, ASC-unmedicated had significantly increased ML rambling compared to ASC-medicated (*t* = 2.491; *p* = 0.012, 95% CI [0.0807, 0.762]). Thus, final reported ANOVAs did not separate ASC group according to medication status.

### Multiple factor analysis (MFA) of postural sway data

FactoInvestigate (R package) did not identify any outliers in either group, or in any of the four sway conditions. Based on Horn’s parallel test, Dimensions 1 and 2 were retained for interpretation. When a separate MFA was conducted on each group individually (ASC group and NC group), the same sway variables loaded onto the Dimension 1 and Dimension 2 across both groups. Dimension 1 was represented by peripheral nervous system, fast timescale fluctuations in the CoP signal: AP trembling, ML trembling, and sway path were all highly correlated with each other, represented by high positive loadings across the 4 sway conditions (both groups with loadings ≥ 0.40). On the other hand, Dimension 2 was represented by central nervous system, slow timescale fluctuations in the CoP signal: AP rambling, ML rambling, and sway area were highly correlated and mapped onto Dimension 2 more strongly than Dimension 1 (both groups with loadings ≥ 0.40). See Supplementary Materials for group-specific factor loadings on both dimensions.

**Fig. 2:**
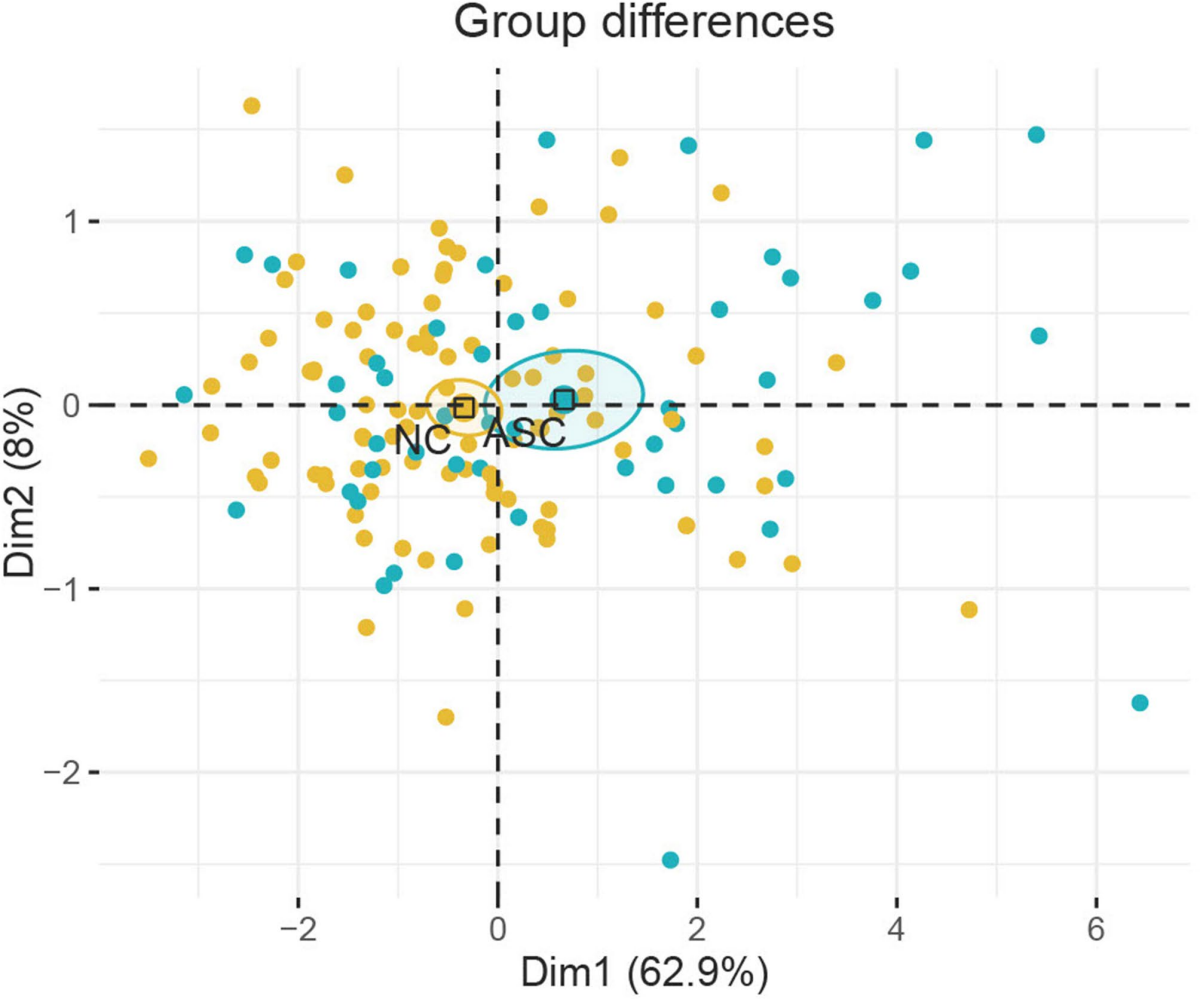
Group differences on Dimension 1 and Dimension 2 across ASC and NC groups. Group differences on Dimension 1 and Dimension 2 across ASC (in blue), and NC (in yellow) groups. Visually, we observe a greater proportion of the variance in sway is due to Dimension 1 in the ASC group as compared to the NC group, and approximately the same proportion of the variance due to Dimension 2 across both groups

The faster timescale variables of Dimension 1 contributed more strongly to variance in overall postural sway in the ASC group than in the NC group (~ 70% of the variance coming from Dimension 1 in ASC, versus ~ 54% in NC), whereas approximately the same contributions to variance were observed for Dimension 2 for both groups (~ 8%). Moreover, in the combined MFA, the group difference in Dimension 1 was confirmed significantly different between ASC and NC (*p* = 0.003). See Fig. 2. The four sway conditions were similar in terms of the specific sway metrics that mapped onto each dimension in each group.

**Fig. 3:**
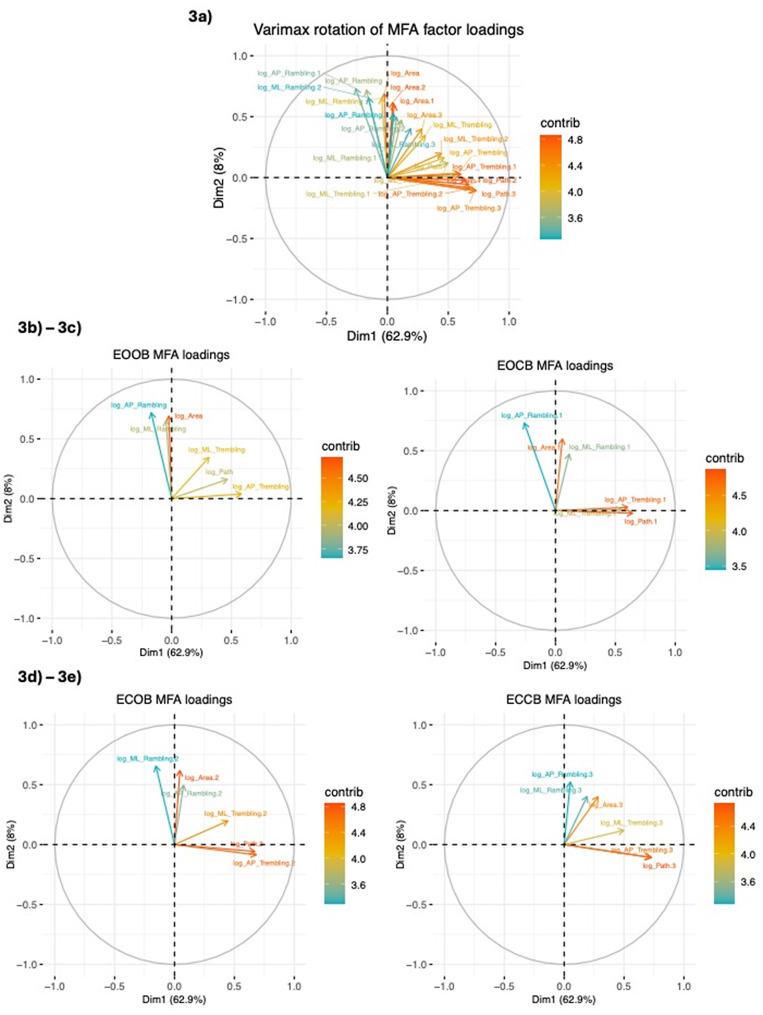
(**3a-3e**). MFA Varimax Rotated Loadings for Dimensions 1 and 2. In **3a**, MFA loadings for Dimensions 1 and 2 (when groups are combined, for all 4 conditions). In **3b-e**, MFA loadings for each condition (EOOB, EOCB, ECOB, ECCB, respectively) shown separately. The contributions (“contrib”) for each variable are represented by color, with red/orange indicating highest contributions to that sway condition and blue/green representing lowest. In the figure, variables denoted by “long” map onto slow timescale sway processes, whereas those denoted by “short” are fast timescale sway processes

A biplot of rotated loadings for all 24 variables for the combined MFA can be seen in Fig. 3, with the color of each arrowed line indicating the contribution for that variable. Warmer colors, such as red and orange, indicate more robust representation of a particular variable on the dimensions. For clarity, Fig. 3b and e show separate biplots for each of the 4 conditions that are depicted together in 3a. Examination of these biplots suggest that manipulations of sensory conditions (across the 4 sway conditions) did not strongly alter loadings for any of the measures.

## Discussion

The current study identified clear differences in adults with autism on a uniquely comprehensive set of postural sway metrics. First, we observed significantly increased sway path and ML trembling in ASC compared to NC, and trending increases in AP rambling and sway area. After applying Bonferroni correction, group by vision interactions remained significant between ASC and NC on sway area and AP rambling. Additionally, using data-driven MFA on metrics derived from these area, path, and rambling-trembling analytic approaches, we observed a significantly greater proportion of variance in sway in the ASC group was accounted for by fast-timescale sway metrics compared to in the NC group. Slow-timescale sway metrics accounted for a similar proportion of variance across the two groups. The fast-timescale sway metrics, including sway path and trembling, are thought to represent peripheral nervous system processes of proprioception [34]. Importantly, our findings suggest that increased postural sway in adults with autism is predominantly associated with differences in proprioception-driven corrections. This is consistent with the larger effect sizes of group differences for the metrics composing the fast dimension from the MFA, as well as significantly increased sway path and ML trembling in ASC compared to NC.

Sway directionality differences among the two groups were not specified in our hypotheses, as the existing literature is mixed, showing differences in sway in both AP and ML in autistic individuals [6, 24, 68, 69]. Our findings align with previous reports of increased sway area and sway path in autism [16, 70], as well as increased rambling [68]. Stania et al. [68] did not find trembling differences in their sample of autistic children, while our analyses highlighted increased trembling in ASC. However, it is important to note that the current study investigated a different age group (adults rather than children with autism), and our sample size was larger (49 ASC compared to 16).

In exploratory analyses of the interaction between group and sway condition (eyes open or closed), some noteworthy patterns emerge in the postural sway data. Interestingly, parametric tests indicated that ASC sway deficits are more apparent in the eyes open conditions in several metrics (sway area, AP rambling, and AP trembling) while sway deficits were less commonly observed for open base conditions. These interactions may also indicate that sensory input, particularly visual, has a greater influence on postural sway in autistic adults than base of support of the feet (somatosensory/vestibular component). Another potential interpretation to consider is that ASC may engage in sensory reweighting of visual input (i.e., less reliance on visual information) compared to NC participants. These interpretations suggest a possible role for visual attention moderating or influencing sway performance in autistic adults. It is also possible that the exacerbated sway during eyes-open in ASC could be partially explained by difficulties with multi-sensory integration (i.e. one’s ability to integrate multiple sensory inputs and modalities from the environment to efficiently create coherent models of the world [71]). Past work has shown that individuals with autism often have reduced multi-sensory integration abilities [72–74] and introducing additional sensory demands to a sway task often is associated with increases in postural sway [75, 76]. In other words, when there is increased visual sensory information, it may become more challenging to maintain selective attention to the balance task due to these proposed integration difficulties in ASC. However, more research will need to be conducted to specifically explore these theories of visual attention and the role of multi-sensory integration in postural sway in adults with autism.

In summary, by integrating complimentary methods to measure of postural sway, the current study provides a more consistent picture of the sway alterations in ASC that can inform investigations of associated neural systems in the future.

### Limitations and future directions

The present study has several limitations. First, the four sway conditions (EOOB, EOCB, ECOB, ECCB) were not randomized due to limitations of the original study design, so they were always measured in all participants in the same order. This has the benefit of maintaining a constant order of conditions among participants. However, this also may have influenced levels of sway in the later-presented conditions due to increasing fatigue in participants, or in earlier-presented conditions if participants had difficulties in adjusting to the task. Additionally, there were significant group differences on body weight and race, and the inclusion of weight and race as covariances accounted for a high degree of variance in most sway metrics. Future studies should make increased efforts to match participants based on weight, BMI, and race.

Though the current study provides compelling evidence that postural sway aberrations are demonstrated in a cohort of adults with ASC, the range of ages in this study are somewhat limited, as most of our participants were in young adulthood, specifically in the 20–30-year-old age range. Critically, future studies should include a wider range of ages in adulthood and/or do a replication study on older adults with autism in midlife and elderly life stages (~ 40–80 years old) to elucidate how the aging process is related to postural sway deviations in autism.

Finally, diagnostic records and clinical diagnosis of autism were not obtained in all participants, though recent research suggests that motor-based differences persist in adults without a diagnosis who score high on the Broad Autism Phenotype, a measure of autistic traits in the general population [38]. Replication studies may want to take a more stringent approach to inclusion criteria by assessing all participants with “gold-standard” diagnosis techniques such as ADOS-2 administration to examine effects of condition severity. Another limitation of the current study is that measures of autism symptom severity were not included. Considering previous findings identifying significant associations between increased postural sway and greater severity of autism symptoms, it will also be important to further examine the associations of current sway findings and autism symptoms in future studies.

On the other hand, considering the RDoC criteria promoted by recent initiatives of the NIMH [77], it may also be a strength of the present work that it was less reliant on diagnostic boundaries, especially in the adult population where it becomes even harder to obtain a valid clinical diagnosis of autism with the relative treatment and research gap in adults compared with youth with autism [78]. Importantly, the discovery of objective biomarkers may aid in future diagnosis of autism throughout the lifespan and documented and well-categorized alterations or impairments in postural stability processes may advance these efforts. Though some of these efforts in creating motor-based biomarkers to use in future diagnosis of autism have been put forth in other recent studies such as Wu et al. [79], research in this domain is still in relative infancy.

While this work may reveal preliminary nervous system contributions in autism, determining if these findings are autism-specific is challenging due to the high rates of psychotropic medication use and psychiatric comorbidities in autistic adult samples [49, 50, 80, 81]. Nonetheless, the present study adds to the literature by further specifying postural sway aberrations found in adults with autism, and points to potential nervous system mechanisms underlying these differences.

## Conclusion

The current study brings together traditional approaches and rambling-trembling decomposition to measure postural sway in adults on the autism spectrum. The use of these measurement techniques in combination with one another, as well as their visualization using MFA, offer key insights into the mechanisms of sway and point to differential origins in sway differences in both the neurotypical and neurodiverse populations.

The current study replicates previous findings of disturbed postural sway and autism, such that postural sway was found to be increased in several metrics in adults on the autism spectrum compared to neurotypical comparison participants. Moreover, in both groups, most of the variability in postural sway was predominantly captured by processes associated with the peripheral nervous system and proprioception as opposed to the central nervous system, and this difference was seen to a greater extent in the ASC group. These findings contribute to understanding how peripheral and central nervous system mechanisms may shape balance control in autism, and may motivate future studies to identify the neural substrates of sway alterations in this condition.

## Supplementary Information

Below is the link to the electronic supplementary material.

**Supplementary Material 1**: See “Supplemental Materials_SwayManuscript.docx” for supplementary tables and figures on MFA findings and ASC group clinical characterization.

## Data Availability

No datasets were generated or analysed during the current study.
