# Supplementary material for: Postural sway dynamics in adults across the autism spectrum: a multifactor approach
Source: Mol Autism. 2025 Aug 28;16:44. doi: 10.1186/s13229-025-00676-y (PMC12395646; doi:10.1186/s13229-025-00676-y)
Supplement: Supplementary file 1 — Supplementary Material 1: See “Supplemental Materials_SwayManuscript.docx” for supplementary tables and figures on MFA findings and ASC group clinical characterization. [file 13229_2025_676_MOESM1_ESM.docx]

**Supplemental Materials**

**Table 4. Characterization of ASC group - Co-occurring psychiatric disorders**

| **DSM-V Classification** | ***n* (% out of 28)** |
| --- | --- |
| Depressive disorders | 22 (78.57%) |
| Anxiety disorders | 22 (78.57%) |
| ADHD | 5 (17.86%) |
| Psychosis disorders | 0 (0%) |
| Bipolar disorder* | 2 (7.14%) |
| Substance use disorders | 3 (10.71%) |
| Alcohol use disorder* | 1 (3.57%) |
| Traumatic/stressor related disorders | 4 (14.29%) |
| Multiple classes | 22 (78.57%) |

**Note** Out of 49 total in the ASC group, *n*=28 had at least 1 co-occurring psychiatric disorder at the time of testing. *n*=22 total met criteria for a current/past depressive disorder (12 current, 10 past); *n* = 16 met criteria for Major Depressive Disorder (MDD). *n=*22 total met criteria for a current/past anxiety disorder (14 current, 8 past): *n*=6 met criteria for Generalized Anxiety Disorder (GAD), *n*=5 met criteria for Social Anxiety Disorder (SAD), *n*=2 met criteria for Specific Phobia, *n*=1 met criteria for Panic Disorder, and *n*=8 met criteria for 2 or more anxiety disorders. *n=*5 met criteria for ADHD, but 2 additional ASC participants had been previously diagnosed but did not meet SCID-V criteria at the time of testing. No participants met criteria for a psychosis spectrum disorder. *n=*3 met criteria for past mild to moderate Substance Use Disorder (SUD), and n=1 met for criteria for current mild Alcohol Use Disorder (AUD).

* *n=*4 met criteria for current/past (2 current, 2 past) Post-Traumatic Stress Disorder (PTSD). Of 28 total ASC participants meeting criteria for at least 1 co-occurring psychiatric condition current or past, 22 participants (78.6%) met criteria for 2 or more co-occurring psychiatric disorders in multiple categories.

.**n=*2 met criteria for current/past Bipolar Disorder (*n* = 1 Type I and *n =* 1 Type 2): these participants were included after determining the Bipolar Disorder diagnosis was well-managed with medication or other forms of treatment at the time of testing.

**n*=1 met criteria for current mild Alcohol Use Disorder, and was included as current alcohol use was not clinically impairing functioning in any major life domains above and beyond ASC diagnosis

**Table 5. Characterization of ASC group – Scores on autistic trait questionnaires**

| **Measure** | | ***M*** | ***SD*** |
| --- | --- | --- | --- |
| AQ-Full | Attention to detail subscale (sum) | 6.78 | 1.95 |
|  | Attention switching subscale (sum) | 7.84 | 1.75 |
|  | Communication subscale (sum) | 7.33 | 2.25 |
|  | Imagination subscale (sum) | 4.65 | 2.80 |
|  | Social skills subscale (sum) | 6.59 | 1.70 |
|  | Total (sum) | 33.18 | 6.86 |
| BAP-Q* | Aloof subscale (average) | 3.60 | 0.83 |
|  | Pragmatic language subscale (average) | 3.18 | 0.67 |
|  | Rigidity subscale (average) | 3.38 | 0.80 |
|  | Total | 3.39 | 0.55 |
| SRS | Social Communication & Interaction subscale (sum) | 79.71 | 18.29 |
|  | Repetitive Interests and Restricted Behaviors subscale (sum) | 20.96 | 6.11 |
|  | Total | 100.70 | 18.40 |

**Note** Mean total scores on select self-report measures (Autism Spectrum Quotient, Full - AQ; Broad Autism Phenotype Questionnaire - BAP-Q; Social Responsiveness Scale - SRS). These scales were not repeated in the NC group. Clinical cut-off scores are represented by a clinical threshold of ≥ 32 total and a screening cut-off of ≥ 26 total on the AQ (Baron Cohen et al., 2001), clinical cut-offs of ≥ between 3.0 and 3.75 average item scores for the whole measure and each subscale for the BAP-Q (Hurley et al., 2007), and a cut-off total score of ≥ 75 on the SRS (Constantino, 2005; Constantino, 2012). *Since BAP-Q clinical cut-off scores are referred to in averages per subscale and on the entire measure (Hurley et al., 2007), averages were used rather than total scores

**Figure 4. Percent explained variance from MFA Dimensions 1-5**

**Note.** Percent explained variance from Dimensions 1-5, where Dimensions 1 represents 62.9% of the variance in the sample, Dimension 2 represents 8.0% of the variance, Dimensions 3-5 represent 6%, 4.1%, and 3.3% of the variance respectively

**Figure 5. Average variable correlations for all 4 sway conditions (and Group).**

**Note.** Average variable correlations for all 4 sway conditions (and Group). Most correlations map higher onto Dimension 1 than Dimension 2. The sensory conditions for the MFA were highly correlated. Group was more associated with Dimension 1 than Dimension 2, but was not highly related to either dimension.

**Table 3a. Substantial factor loadings - ASC only**

|  | **Dimension 1 loadings (70.6%)** | | | | **Dimension 2 loadings (8.0%)** | | | |
| --- | --- | --- | --- | --- | --- | --- | --- | --- |
|  | EOOB | EOCB | ECOB | ECCB | EOOB | EOCB | ECOB | ECCB |
| *Sway path* | *.35* | *.49* | *.49* | *.54* | *.25* | *<.10* | *<.10* | *<.10* |
| *AP trembling* | *.47* | *.51* | *.57* | *.59* | *.12* | *<.10* | *<.10* | *<.10* |
| *ML trembling* | *.33* | *.49* | *.45* | *.48* | *.32* | *<.10* | *.14* | *.10* |
| *Sway area* | *.12* | *.24* | *.19* | *.31* | *.64* | *.49* | *.56* | *.42* |
| *AP rambling* | *<.10* | *<.10* | *.16* | *.15* | *.59* | *.60* | *.47* | *.56* |
| *ML rambling* | *<.10* | *.24* | *<.10* | *.24* | *.66* | *.41* | *.67* | *.44* |
| ***** Numbers in black indicate substantial dimension loadings > .40*** | | | | | | | | |

**Table 3b. Substantial factor loadings - NC only**

|  | **Dimension 1 loadings (54.3%)** | | | | **Dimension 2 loadings (8.7%)** | | | |
| --- | --- | --- | --- | --- | --- | --- | --- | --- |
|  | EOOB | EOCB | ECOB | ECCB | EOOB | EOCB | ECOB | ECCB |
| *Sway path* | *.38* | *.42* | *.47* | *.47* | *.16* | *<.10* | *<.10* | *<.10* |
| *AP trembling* | *.52* | *.49* | *.53* | *.52* | *<.10* | *<.10* | *<.10* | *<.10* |
| *ML trembling* | *.35* | *.38* | *.37* | *.36* | *.31* | *<.10* | *.27* | *.24* |
| *Sway area* | *.19* | *.15* | *.17* | *.27* | *.54* | *.55* | *.57* | *.47* |
| *AP rambling* | *<.10* | *<.10* | *.15* | *.12* | *.59* | *.57* | *.50* | *.53* |
| *ML rambling* | *.15* | *.16* | *<.10* | *.17* | *.50* | *.47* | *.49* | *.45* |
| ***** Numbers in black indicate substantial dimension loadings > .40*** | | | | | | | | |

**Note.** Substantial MFA factor loadings for ASC only (Table 3a), and NC only (Table 3b). Numbers in black in each table indicate substantial dimension loadings *>* .40. In both ASC and NC groups, sway path, AP trembling, and ML trembling consistently loaded onto Dimension 1, and sway area, AP rambling, and ML rambling consistently loaded onto Dimension 2. In both groups, Dimension 1 accounted for more overall variance in sway than Dimension 2. However, Dimension 1 accounted for more variance in sway in ASC (70.6%) compared to NC (54.3%).
